# miR-21 and miR-155 are associated with mitotic activity and lesion depth of borderline melanocytic lesions

**DOI:** 10.1038/bjc.2011.288

**Published:** 2011-08-23

**Authors:** V Grignol, E T Fairchild, J M Zimmerer, G B Lesinski, M J Walker, C M Magro, J E Kacher, V I Karpa, J Clark, G Nuovo, A Lehman, S Volinia, D M Agnese, C M Croce, W E Carson

**Affiliations:** 1 Department of Surgery, The Ohio State University Comprehensive Cancer Center, N924 Doan Hall, 410 West 10th Avenue, Columbus, OH 43210, USA; 2 Integrated Biomedical Sciences Program, The Ohio State University Comprehensive Cancer Center, Columbus, OH 43210, USA; 3 Human Cancer Genetics Program, Department of Molecular Virology and Medical Genetics, The Ohio State University Comprehensive Cancer Center, Columbus, OH 43210, USA; 4 Department of Medical Oncology, The Ohio State University Comprehensive Cancer Center, Columbus, OH 43210, USA; 5 Department of Pathology and Laboratory Medicine, Weill Medical College of Cornell University, New York, NY 1002, USA; 6 Department of Oral Pathology at the New York Hospital Queens, New York, NY 10016, USA; 7 Department of Pathology, The Ohio State University Comprehensive Cancer Center, Columbus, OH 43210, USA; 8 Center for Biostastistics, The Ohio State University Comprehensive Cancer Center, Columbus, OH 43210, USA

**Keywords:** malignant melanoma, melanocytic proliferations, microRNA

## Abstract

**Background:**

Expression of microRNAs (miRs) has been shown to be altered in many solid tumours and is being explored in melanoma. The malignant potential of some melanocytic lesions is difficult to predict. We hypothesised that characterisation of miR expression in borderline melanocytic proliferations would lead to the identification of a molecular profile that could be used with known prognostic factors to differentiate lesions with high malignant potential.

**Methods:**

The miR expression profile of melanocytic lesions (benign naevi, malignant melanoma and borderline melanocytic tumours) was evaluated by real-time PCR.

**Results:**

PCR analysis revealed primary cutaneous melanomas had an 8.6-fold overexpression of miR-21 and a 7.5-fold overexpression of miR-155 compared with benign naevi (*P*<0.0001). *In situ* hybridisation confirmed these results. miR-21 and miR-155 were significantly overexpressed within borderline lesions (*P*=0.0011 and *P*=0.0048, respectively). When borderline lesions were categorised by mitotic activity and Breslow thickness, miR-21 was associated with mitotic activity and miR-155 was associated with thickness (*P*<0.025). Among 14 patients with borderline lesions who underwent sentinel lymph node biopsy (SLNB), positive SLNB was associated with increased miR-21 and miR-155 in the primary lesion compared with lesions with a negative SLNB.

**Conclusion:**

MicroRNA expression profiles can be used to characterise atypical melanocytic lesions.

MicroRNAs (miRs) are a recently discovered class of small non-coding RNAs (Zeng, 2006). The binding of miRs to target mRNAs inhibits gene expression at the protein level. MicroRNAs are synthesised as long primary transcripts in the nucleus to produce RNA precursor molecules of ∼70 nucleotides in length (pre-miRs). The pre-miRs are then exported to the cytoplasm and processed to form double-stranded RNA that measures 21–23 nucleotides in length. One strand of the duplex is selectively stabilised and base pairs with its complementary mRNA, thus inducing mRNA degradation (Hutvagner and Zamore, 2002). Alternatively, some miRs inhibit the initiation of translation or interfere with translation already in progress by causing the ribosome to disengage from the target mRNA (Engels and Hutvagner, 2006).

Each miR is believed to regulate multiple genes. Hundreds of miR genes have been identified in humans, and thus the regulatory potential of miRs is enormous (Conrad *et al*, 2006). Several groups have shown that miRs may act as key regulators of multiple cellular processes. MicroRNAs are dysregulated in most forms of cancer and have a role in maintaining the malignant phenotype via alterations in processes such as cellular adhesion, angiogenesis, cell cycle control and apoptosis (Lu *et al*, 2005; Dalmay and Edwards, 2006).

Melanocytic proliferations are among the most diversified of cutaneous lesions ranging from completely benign naevi such as the junctional naevus, congenital naevus and blue naevus to those with overtly malignant features. This group of proliferations is nebulous both clinically and morphologically. These so-called borderline (also referred to as indeterminate) melanocytic lesions manifest architectural features and cytologic atypia, which exceeds those encountered in naevi. In such cases, the potential for an aggressive biological course arises. A categorical approach to the borderline melanocytic proliferation has been proposed by Crowson *et al* (2001) wherein they recognised four main categories; (1) borderline melanocytic proliferations arising in association with a deep penetrating naevus, (2) those exhibiting borderline features of nevoid melanoma, (3) atypical Spitz tumours and (4) pigmented epithelioid melanocytoma formerly designated animal-type melanoma. In addition, dysplastic naevi may also pose a diagnostic dilemma to clinicians (Barnhill *et al*, 2003). Importantly, there do exist alternate classification systems that capture the complexity and variability inherent in this class of melanocytic lesions (Barnhill *et al*, 2010); however, it was elected to proceed under the auspices of the classification scheme that was in use at our institution.

Borderline lesions pose a therapeutic dilemma for clinicians, as a false-negative reading of a benign naevus could lead the clinician to recommend against further therapy. Under-treatment of a melanocytic lesion with malignant potential could thus adversely impact survival. Conversely, over-treatment of a false-positive reading of malignancy could lead to needless morbidity from surgery or adjuvant therapy. Therefore, we sought to identify molecular markers that could be used to further characterise these lesions and predict their clinical behaviour. Current prognostic indicators for primary melanoma lesions include Breslow depth and ulceration (Balch *et al*, 2001). These factors and mitotic activity are often considered in the diagnosis of borderline lesions but by themselves do not serve to define such lesions as malignant or benign. We hypothesised that the analysis of miR expression in melanoma tumours would lead to the identification of a molecular profile that would correlate with known prognostic factors (mitotic index and lesion depth) and could be used in conjunction with them to differentiate borderline lesions with high or low malignant potential. This information could also provide insight into the role played by miRs in the development and progression of malignant melanoma.

## Patients and methods

### Patient samples

A series of borderline melanocytic lesions, dysplastic naevi, benign dermal naevi and melanomas were evaluated and diagnosed by one of the authors of this paper (CMM). The melanomas were classified according to established criteria with recognition of the presence or absence of the radial growth phase and/or vertical growth phase. The cases were derived retrospectively and reflect consecutive encounters from the routine and consultative dermatopathology practice of the Ohio State University Medical Center's Department of Pathology. The cases were encountered between 2000 and 2006 (IRB no. 2007 C0015). For RNA isolation, four to eight unstained 20 *μ*m sections were cut from formalin-fixed, paraffin-embedded tissue derived from punch biopsies.

### Isolation of total RNA

Paraffin-embedded tissue was harvested utilising the RecoverAll Total Nucleic Acid Isolation Kit as per the manufacturer's recommendations (Ambion, Foster City, CA, USA). Nuclease-free water was used to elute the RNA.

### Real-time PCR

Single tube TaqMan MicroRNA Assays were used to detect and quantify mature miRs. All reagents, primers and probes were obtained from Applied Biosystems (Foster City, CA, USA). PCR for RNU6B, a small ubiquitous RNA, was used to normalise all RNA samples. Reverse transcriptase reactions and real-time PCR were performed according to the manufacturer's protocols. Gene expression levels were quantified using the ABI Prism 7900HT Sequence Detection System (Applied Biosystems). Comparative real-time PCR, using the Ct method, was performed in triplicate, including no-template controls (Schmittgen *et al*, 2008). Expression was calculated as fold change (2^−ΔCT^) compared with internal control RNU6B.

### *In situ* hybridisation

The protocol for detection of miRs in paraffin-embedded tissue by *in situ* hybridisation has been previously published (Nuovo, 2008). The sequence of the locked nucleic acid modified cDNA probes were: miR-155=5′-ACCCCTATCACGATTAGCATTAA-3′ and miR-21=5′-TCAACAGTCAGTCTGATAAGCTA-3′. The probes were labelled with the 3′oligonucleotide tailing kit using biotin as the reporter (Enzo Diagnostics, Farmingdale, NY, USA). Negative controls were omission of the probe, use of a scrambled probe and internal controls present in the tissue.

### REMARK considerations

This study adhered to the 20 distinct REMARK criteria for the validation and reporting of tumour marker studies except that outcome determination was not a primary aim of this study. As this study examined the association of miR expression with standard prognostic variables, and not recurrence or time to clinical event, multivariate analyses were not performed (McShane *et al*, 2005).

### Statistical analysis

For the comparisons of miR expression between benign naevi and malignant melanoma cases, summary statistics for the fold change (mean, median, s.d., minimum and maximum values) were first calculated for benign naevi and malignant melanoma separately. Two sample *t*-tests using the −ΔCT values were performed; Holm's method was applied to adjust for multiplicity and control the overall family-wise type I error rate at *α*=0.05. Similarly, expression of miR-21, miR-155 and miR-211 among five types of indeterminate lesions *vs* benign naevi, were performed on the −ΔCT values using analysis of variance. Each type's miR expression was compared with benign naevi, and Dunnett's method was used to adjust the *P*-values. Nonparametric Wilcoxon rank-sum tests were used to compare miR-155 and miR-21 expression between lesions with and without mitotic activity >1 in 10 HPF, lesions with a depth <1 *vs* ⩾1 mm, and positive sentinel lymph node biopsy (SLNB) (yes *vs* no) using an adjusted *α*=0.025 level of significance to control for the two miR comparisons for each parameter.

## Results

### Comparison of miR-21 and miR-155 expression levels in benign naevi and malignant melanoma

**Figure 1:**
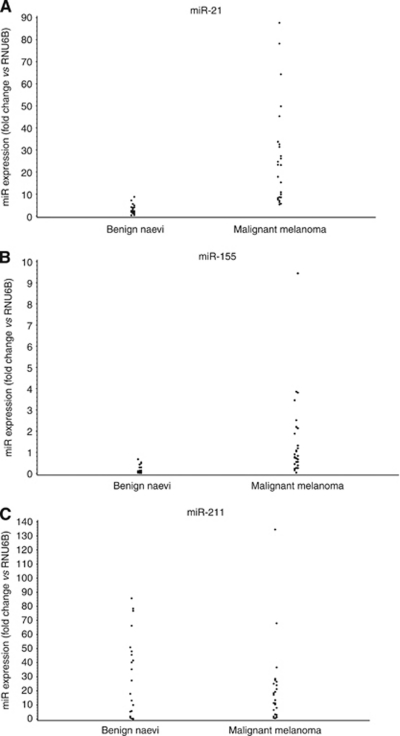
Real-time PCR was used to determine the expression of (**A**) miR-21, (**B**) miR-155 and (**C**) miR-211 in benign naevi (*n*=22) and malignant melanoma (*n*=28) tissue samples. miR-21 and miR-155 were significantly upregulated (*P*<0.0001) and miR-211 was downregulated (*P*=0.5474). Data were expressed as fold change relative to RNU6B.

Previously, our group evaluated the expression of 224 mature miRs by microarray in solid cancers (Volinia *et al*, 2006, 2010). Several miRNAs were found to be preferentially upregulated in melanoma when compared with corresponding normal tissues (miR-17-5p, miR-21, miR-107, miR-130, miR-155, miR-181b and miR-221). In this study, total RNA was isolated from paraffin-embedded tissue (Supplementary Tables S1 and S2) and tested for the expression of these miRs by real-time PCR (Figure 1A and B). Using the small nuclear RNA RNU6B as a control, miR-21 and miR-155 were expressed to a significantly greater level in malignant melanoma samples (fold change *vs* RNU6B=25.9±22.4 and 1.5±1.9, respectively) as compared with benign naevi (fold change 3±2.1 and 0.2±0.2, respectively, *P*<0.0001). Significant overexpression of four of the other miRs was also observed (fold change=2.6±1.6, 0.15±0.16, 0.8±0.5 and 6.3±4.3 for miR-17-5p, miR-107, miR-130a and miR-181b, respectively) compared with benign naevi (fold change=0.6±0.7, 0.03±0.03, 0.3±0.3 and 1.5±1.6 for miR-17-5p, miR-107, miR-130a and miR-181b, respectively, *P*<0.0001, Supplementary Figures 1A–D). Analysis of miR expression levels in malignant melanoma compared with benign naevi revealed miR-21 and miR-155 to be the most highly upregulated (average fold increase 7.8, 95% CI: 5.1, 11.9), and therefore these miR's were chosen for further evaluation. miR-211 was previously identified by genome wide array CGH as being downregulated in solid cancers, including melanoma (Volinia *et al*, 2010). miR-211 also was downregulated in our data set when measured by real-time PCR (fold change *vs* RNU6B was 20.4±27.5 in malignant melanoma *vs* 29.6±28.8 in benign naevi), however, this finding was not significant (*P*=0.5474) (Figure 1C). Examination of miR-21 and miR-155 expression in melanomas with differing growth phases revealed a nonsignificant trend towards increased miR-155 and miR-21 expression in melanomas exhibiting an incipient or fully evolved vertical growth phase as compared with melanomas with only a radial growth phase (data not shown).

### *In situ* hybridisation demonstrates the presence of miR-21 and miR-155 within melanoma tumours

**Figure 2:**
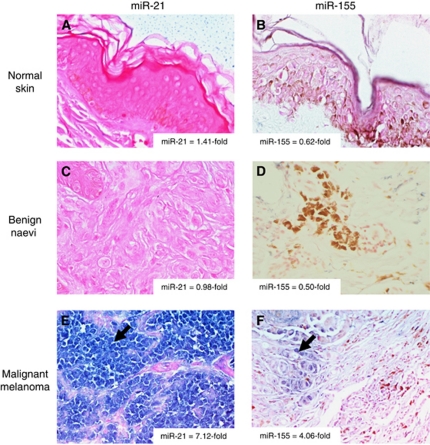
*In situ* hybridisation for miR-21 and miR-155 in (**A** and **B**) normal skin (200 × ), (**C** and **D**) benign naevi (400 × ) and (**E** and **F**) malignant melanoma (400 × ). Specific staining for miR of interest is blue (arrow) and counterstain is pink, some samples contain melanin pigment (brown coloration). Validation of miR-21 and miR-155 expression as determined by real-time PCR is indicated in the corner of each panel. Data shown are representative of *n*=4 samples for each tissue type.

In order to confirm the results of real-time PCR, *in situ* hybridisation was used to evaluate the expression of miR-21 and miR-155 in a subset of samples. miR-21 and miR-155 expression was not detected in normal skin (Figure 2A and B – note presence of pink counterstain and lack of blue miR staining) or benign dermal naevi (Figure 2C and D – note pink counter stain). However, miR-21 and miR-155 signals were abundant in malignant melanoma samples (Figure 2E and F – note blue stain). Notably, staining for these miRs was confined to malignant cells, while surrounding normal cells showed no evidence of miR-21 or miR-155 expression. Real-time PCR performed on RNA isolated from the identical tissue samples validated the expression of these miRs (Figure 2A–F– inset).

### Levels of miR-21 and miR-155 in dysplastic naevi and borderline melanocytic lesions

**Figure 3:**
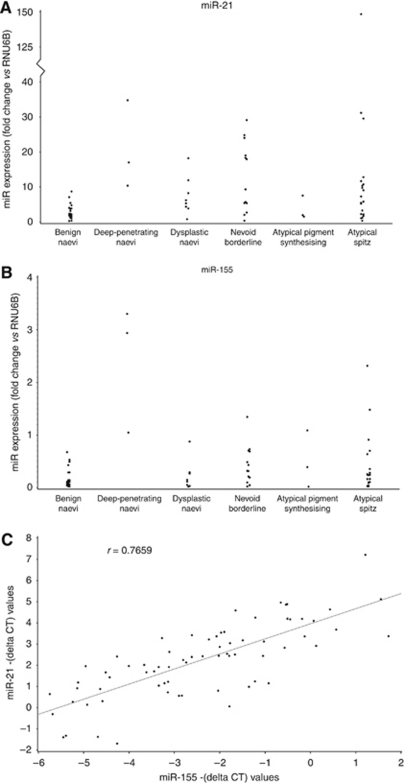
Real-time PCR was used to determine the expression of (**A**) miR-21 and (**B**) miR-155 in several types of borderline melanocytic lesions. Data were expressed as fold change relative to RNU6B. When miR-21 and miR-155 expression levels within individual lesions were plotted against each other a significant positive correlation between the expression of miR-21 and the expression of miR-155 was observed in borderline melanocytic lesions (*P*<0.001, *r*=0.77 (**C**).

To characterise the expression of miR-21, miR-155 and miR-211 in indeterminate lesions, total RNA was harvested from a series of 49 borderline melanocytic lesions and dysplastic naevi (Supplementary Table S1). The samples were analysed for miR expression by real-time PCR. miR-21 and miR-155 expression levels were variable among individual specimens (range=0.3–148.4 fold change *vs* RNU6B for miR-21 and range=0.02–3.31 fold change for miR-155; Figure 3A and B). When expression levels in borderline melanocytic lesions as a group were compared with expression levels in benign naevi (to give a ratio of fold change), miR-21 (ratio of fold change=2.92, 95% CI: 1.56, 5.46; *P*=0.0011) and miR-155 (ratio of fold change=2.67, 95% CI: 1.36, 5.24; *P*=0.0048) were significantly overexpressed. Interestingly, a statistically significant positive correlation between the expression of miR-21 and the expression of miR-155 was observed in borderline melanocytic lesions (*P*<0.001, *R*=0.77, Figure 3C). This relationship was not observed in primary melanoma tumours and suggested that there is coordinate expression of these two miRs in borderline melanocytic lesions.

MicroRNA expression was further analysed according to lesion type in the two largest groups of borderline lesions as compared with benign naevi (ratio of fold change). The nevoid borderline lesions (*n*=13) and the atypical Spitz tumours (*n*=22) manifested significantly increased levels of miR-21 (ratio for nevoid borderline lesion=3.37, 95% CI: 1.24, 9.18; *P*=0.0109; ratio for atypical Spitz lesion=2.68, 95% CI: 1.12, 6.42; *P*=0.0206). miR-155 was upregulated in both the nevoid borderline lesions and atypical Spitz tumours; however, the increase was not significant (*P*=0.1810 and *P*=0.4322, respectively).

### The expression of miR-21 and miR-155 in indeterminate lesions correlates with mitotic activity and lesion depth

**Figure 4:**
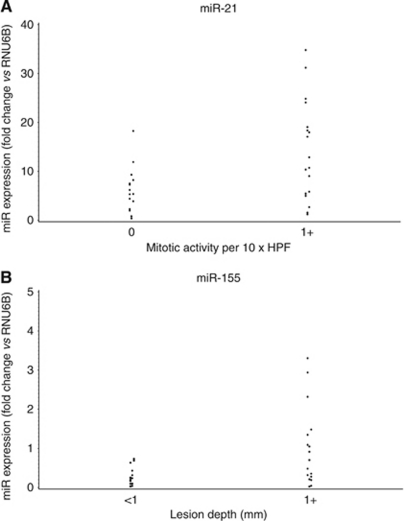
Melanocytic lesions with a mitotic activity >1 in 10 HPF (*n*=19) had significantly higher levels of (**A**) miR-21 expression levels (*P*=0.0227) as compared with lesions with no mitotic activity (*n*=15). Melanocytic lesions with a depth ⩾1 mm (*n*=17) had significantly greater expression of (**B**) miR-155 (*P*=0.0068) as compared with lesions with a depth <1 mm (*n*=17). Data were expressed as fold change relative to RNU6B.

Expression of miR-21 and miR-155 in the entire group of pathologically borderline melanocytic lesions was evaluated in the context of two markers of malignant potential, mitotic activity and depth of invasion. Melanocytic lesions with ⩾1 mitoses per 10 high-power field (*n*=19) expressed significantly higher levels of miR-21 (using RNU6B as a control) as compared with lesions with no mitotic activity (*n*=15) (*P*=0.0227, Figure 4A). This finding goes along with recent papers that show a role for miR-21 in the induction of cancer cell proliferation (Zhang *et al*, 2008; Hatley *et al*, 2010). Given the use of lesion depth >1 mm as a clinical indication for SLNB, the correlation between tumour depth and miR expression was next examined. Borderline melanocytic lesions with a depth ⩾1 mm (*n*=17, range: 1.18–3.1 mm; median depth: 1.68 mm) had significantly higher levels of miR-155 expression as compared with lesions with a depth <1 mm (*n*=17, range: 0.2–0.94 mm; median depth: 0.62 mm) (*P*=0.0068; Figure 4B).

### miR-21 and miR-155 expression levels in specimens from patients who underwent SLNBs

It is difficult to make a definitive determination of malignant potential in some patients with borderline melanocytic lesions, and in some cases the regional lymph nodes will be assessed via SLNBs. In an exploratory analysis, we examined the expression of miR-21 and miR-155 in 14 of the 49 patients who had undergone a SLNB after being diagnosed with a borderline melanocytic lesion. Patients with a positive SLNB were found to have higher levels of miR-21 and miR-155 expression in the primary lesion as compared with lesions from patients who had a negative SLNB. For miR-21 the fold change (*vs* RNU6B) was 15.37±11.37 in SLNB-positive patients *vs* 5.39±4.28 in SLNB-negative patients. For miR-155, the fold change was 0.83±1.09 in SLNB-positive patients *vs* 0.46±0.53 in SLNB-negative patients (Table 1). Although these data suggest a relationship between miR expression and SLN positivity, they did not reach statistical significance (*P*=0.0813, *P*=0.5897 for miR-21 and miR-155, respectively). Plans are underway for a prospective analysis of the expression of miR-21 and miR-155 and other candidate miRs in patients that undergo SLNB for indeterminate lesions.

## Discussion

This study was conducted to determine the relationship between the expression of miRs identified by real-time PCR and factors that contribute to a malignant phenotype in melanocytic lesions. This study found that the expression of miR-21 and miR-155 was significantly higher in primary melanoma lesions and borderline lesions as compared with benign naevi. Also, the expression of miR-21 in borderline melanocytic lesions correlated with mitotic activity, while miR-155 expression correlated positively with lesion depth.

Several studies have examined miR expression in malignant melanoma. A few of these have identified miR-21 and miR-155 as being dysregulated. Using high-resolution array-based comparative genomic hybridisation, Zhang *et al* (2006) showed that 12 of 45 melanoma cell lines had an increased copy number of miR-21 and 7 of 45 melanoma cell lines had an increased copy number of miR-155. They also showed that miR-1-1, miR-96 and miR-296 exhibited an increased copy number in these cell lines; however, these three miRs were not upregulated in our panel of melanoma tumours. Gaur *et al* (2007) characterised the miR profiles of tumours represented in the NCI-60 panel, which contains haematologic, colon, CNS and melanoma cell lines (*n*=8). Using a highly sensitive PCR-based method, they found that miR-146, miR-204 and miR-211 were uniquely expressed in these melanoma cell lines as compared with normal tissues and the other 51 cell lines in the panel. In contrast, we found that miR-211 was downregulated in primary tumours (although not to a significant degree) while the expression of miR-146 and miR-204 was not upregulated. The discrepancy between the two studies can likely be ascribed to the unique make-up of the NCI-60 panel.

A series of recent studies largely agree with the present report. Mueller *et al* (2009) found that miR-21 was upregulated in a panel of highly invasive melanoma cell lines. The recent observation by Levati *et al* (2009) that expression of miR-155 is decreased in a panel of cultured melanoma cell lines runs contrary to our findings and suggest that miR-155 may be differentially regulated in pigmented lesions as compared with cultured cell lines. Importantly, Philippidou *et al* (2010), and Segura *et al* (2010) recently conducted analyses of primary and metastatic melanoma tissue samples and showed miR-155 to be upregulated in these settings. Taken together, these results underscore the fact that miR expression profiles may vary according to the technique employed to measure miR levels, the type of tissue being examined (primary tumour *vs* metastasis), and the applicability of data obtained from cell lines to the clinical situations. Given that overexpression of miR-21 and miR-155 in melanoma primary tumours was confirmed using three distinct assays, we feel that further analysis of these miRs in the setting of melanoma is highly warranted.

Histopathological evaluation is currently the standard method for the classification of borderline lesions. Few studies have examined methods to characterise the malignant potential of borderline melanocytic lesions. Kashani-Sabet *et al* (2009) used a panel of five immunohistochemical markers (ARPC2, FN1, RGS1, SPP1 and WNT2) to distinguish melanoma tumours from benign naevi. In a set of 24 pigmented lesions that were originally misdiagnosed as being either benign or malignant, the pattern of staining of these five markers correctly classified 18 of the 24 lesions (75%). It has been proposed that atypical Spitz tumours represent a distinct subtype of melanocytic lesion, but reliable methods to differentiate between those lesions likely to metastasise and those that will exhibit a benign course have yet to be verified. Immunohistochemical analysis of benign Spitz tumours, atypical Spitz tumours and malignant melanomas for Ki67 (a marker of proliferation), p21 (cell cycle regulator) and fatty acid synthase (metabolic marker) revealed that the atypical lesions possibly represent an intermediate stage (Kapur *et al*, 2005). CD99 was identified by King *et al* (2007) as being present by immunohistochemistry in 15 of 27 (56%) definitively malignant Spitzoid melanomas and only 3 of 58 (3%) benign Spitz tumours. However, the expression of this marker was not explored within borderline atypical Spitz tumours. Analysis of miR expression represents an additional approach to the categorisation of problematic pigmented lesions that someday may be combined with standard immunohistochemical tests.

miR-21 has been shown to be upregulated in a variety of solid malignancies including cancers of the brain, breast, colon, lung, pancreas, prostate, stomach and haematolymphoid tissues, while increased miR-155 expression has been observed in cancers of the thyroid, lung, breast, colon, pancreas and haematolymphoid tissues (Kluiver *et al*, 2006; Roldo *et al*, 2006; Volinia *et al*, 2006; Nikiforova *et al*, 2008). The available data suggest that miRs may function to maintain a malignant phenotype by modulating cell proliferation or the expression of proteins that promote cell survival. Indeed, investigation of primary colorectal cancer lesions from human patients revealed that high miR-21 expression correlated with lymph node metastases, the development of distant metastases and poor survival (Slaby *et al*, 2007; Schetter *et al*, 2008). Functional studies have demonstrated that inhibition of miR-21 in human glioblastoma and breast cancer cell lines led to decreased proliferation and increased apoptosis (Chan *et al*, 2005; Si *et al*, 2007). Other *in vitro* studies have shown that increased miR-21 expression promoted invasion and metastasis and could modulate chemosensitivity (Asangani *et al*, 2008; Blower *et al*, 2008; Zhu *et al*, 2008). Elevated expression of miR-155 in non-small cell lung cancer has also been found to negatively correlate with survival (Yanaihara *et al*, 2006). Aberrant expression of miR-155 under the control of the heavy chain promoter in B cells in a transgenic mouse was sufficient to drive the development of a B-cell malignancy (Costinean *et al*, 2006). In breast cancer, miR-155 overexpression has been linked to increased proliferation and constitutive activation of signal transducer and activator of transcription 3 (Jiang *et al*, 2010) as well as cell survival and chemoresistance through FOXO3a (Kong *et al*, 2010). The pathways by which mature miR-21 and miR-155 become overexpressed and the contributions of this event to disease progression are just now being elucidated (Loffler *et al*, 2007; O'Connell *et al*, 2007; Tili *et al*, 2007; Fujita *et al*, 2008).

We have demonstrated that miR-21 correlates with mitotic activity and miR-155 correlates with lesion depth of borderline lesions. Although SLN-positive borderline lesions showed higher expression of miR-21 and miR-155, additional cases must be examined before any firm conclusions can be drawn. In the future, miR profiles may be utilised to help differentiate between lesions, establish prognosis, and prompt a more thorough search for foci of invasion.

## Figures and Tables

**Table 1:** Expression level of miR-21 and miR-155 in indeterminate melanocytic lesions with sentinel lymph node biopsy

	**Fold change**	
	**miR-21**	**miR-155**
Negative lymph node biopsy	5.7	0.32
	2.06	0.1
	3.28	0.09
	7.54	1.09
	9.32	0.21
	1.98	0.39
	0.38	0.02
	12.86	1.48
Average	5.39±4.28	0.46±0.53
		
Positive lymph node biopsy	19.05	0.43
	9.04	0.36
	10.73	0.16
	1.56	0.02
	17.06	1.05
	34.8	2.94
Average	15.37±11.37	0.83±1.09

Abbreviation: miR=microRNA.
