# Supplementary material for: miR-21 and miR-155 are associated with mitotic activity and lesion depth of borderline melanocytic lesions
Source: Br J Cancer. 2011 Aug 23;105(7):1023–9. doi: 10.1038/bjc.2011.288 (PMC3185929; doi:10.1038/bjc.2011.288)
Supplement: Supplementary Figure 1 [file bjc2011288x1.ppt]

## Slide 1
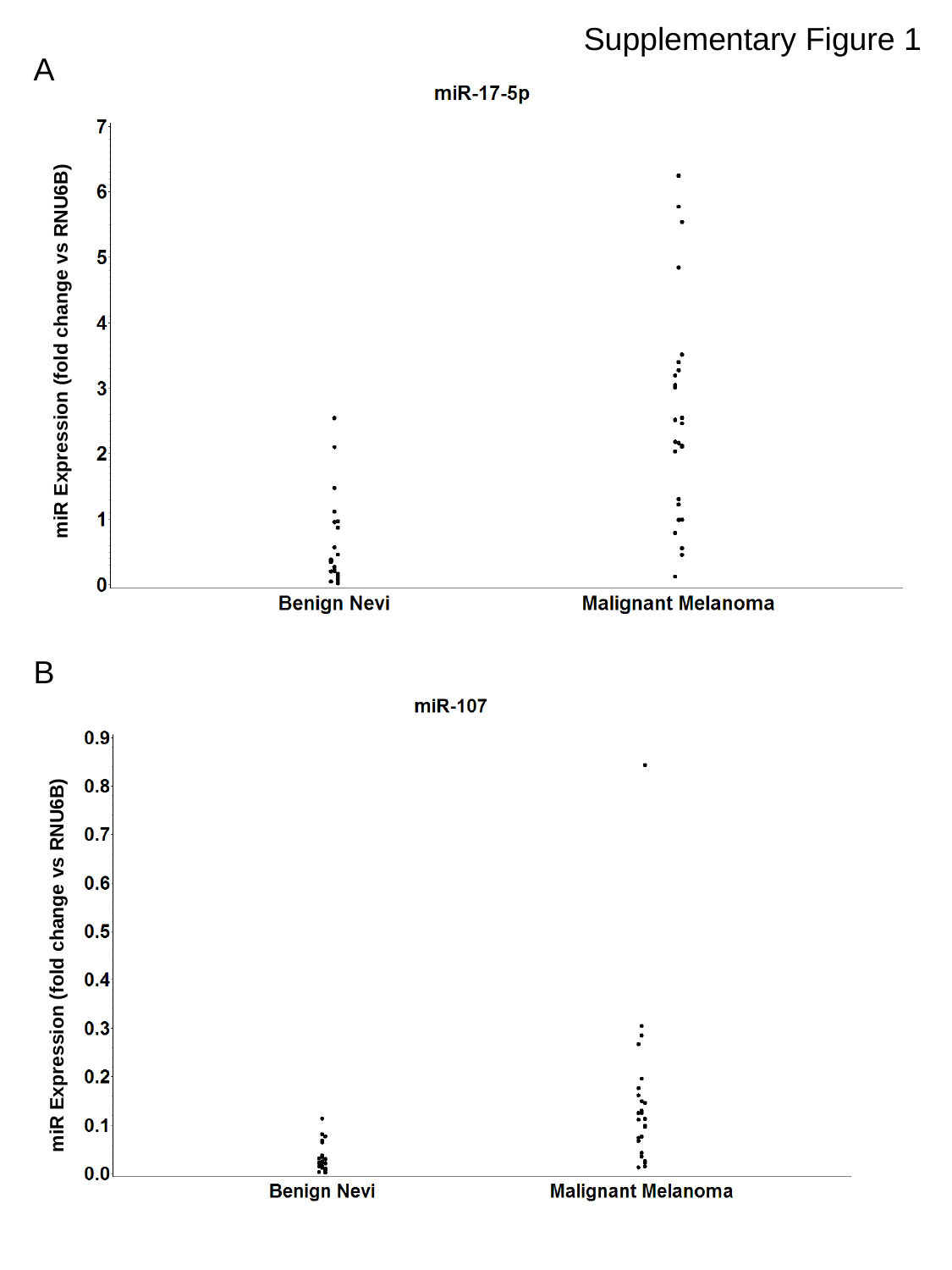

Supplementary Figure 1
A
miR Expression (fold change vs RNU6B)
B
miR Expression (fold change vs RNU6B)

## Slide 2
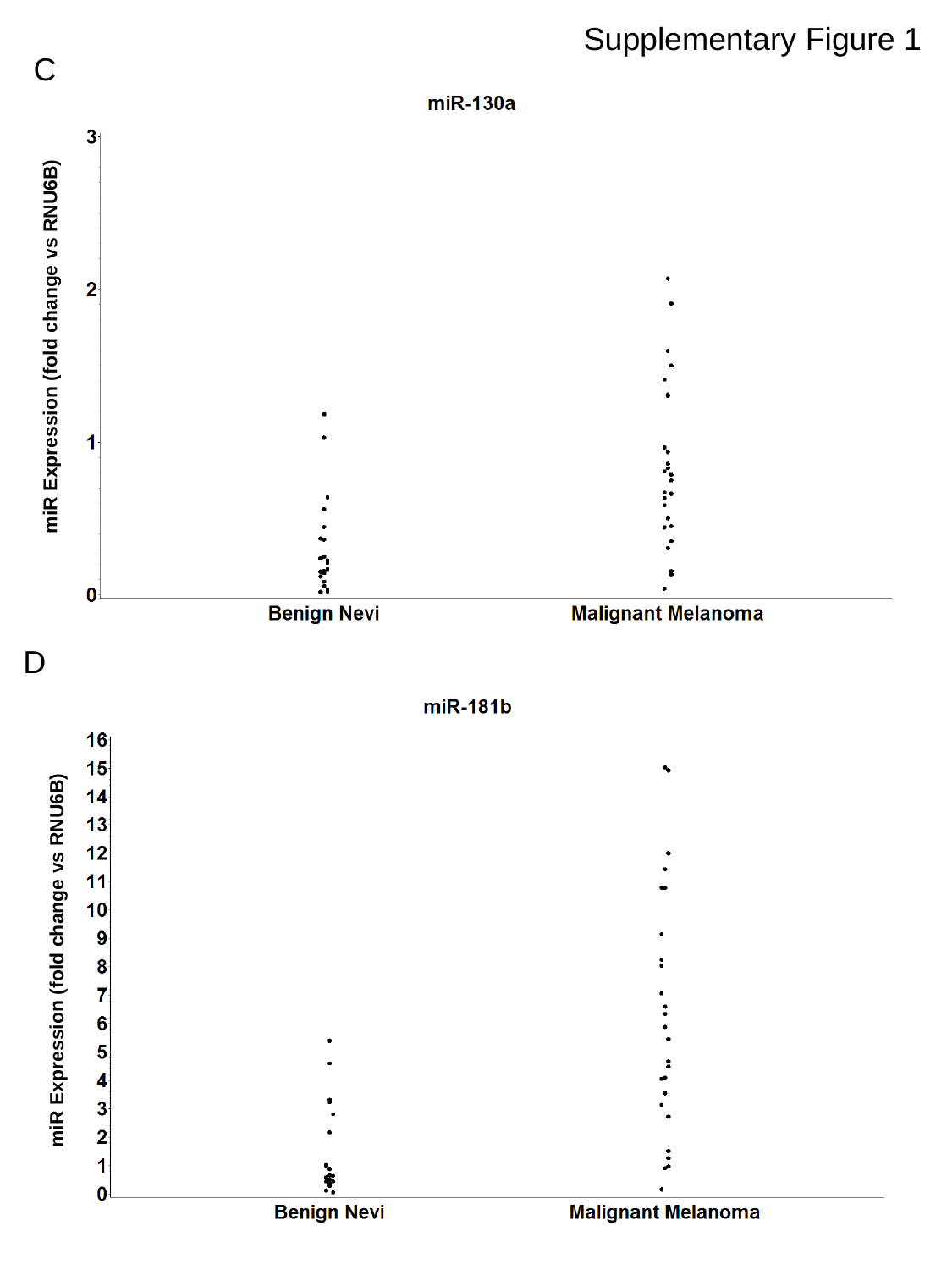

Supplementary Figure 1
C
miR Expression (fold change vs RNU6B)
D
miR Expression (fold change vs RNU6B)
