# Supplementary material for: miR-21 and miR-155 are associated with mitotic activity and lesion depth of borderline melanocytic lesions
Source: Br J Cancer. 2011 Aug 23;105(7):1023–9. doi: 10.1038/bjc.2011.288 (PMC3185929; doi:10.1038/bjc.2011.288)
Supplement: Supplementary Tables 1 and 2 [file bjc2011288x2.doc]

**Supplementary Table 1.** Category, number of lesions and sentinel lymph biopsy (SLNB) availability of patient samples.

| **Type of nevomelanocytic proliferation** | **# in study** | **w/ SLNB** | **Follow-up available** |
| --- | --- | --- | --- |
| Benign nevus | 22 | 0 | 0 |
| Dysplastic nevus | 8 | 0 | 0 |
| Nevoid borderline | 13 | 5 | 6 |
| Atypical Spitz tumor | 22 | 4 | 4 |
| Atypical pigment synthesizing | 3 | 3 | 3 |
| Deep penetrating nevus or overlap features with deep penetrating nevus | 3 | 2 | 2 |
| Malignant melanoma | 28 | 2 | 3 |

**Supplementary Table 2.** Site of malignant melanoma tumors.

| Leg | 10 |
| --- | --- |
| Trunk | 5 |
| Arm | 4 |
| Cheek | 3 |
| Scalp | 2 |
| Ear | 1 |
| Vulva | 1 |
| Unknown | 2 |
